# The complete plastome sequences of nine diploid potato clones

**DOI:** 10.1080/23802359.2021.1883486

**Published:** 2021-03-11

**Authors:** Sai Reddy Achakkagari, Helen H. Tai, Charlotte Davidson, Hielke De Jong, Martina V. Strömvik

**Affiliations:** a Department of Plant Science, McGill University, Sainte-Anne-de-Bellevue, Canada; b Fredericton Research and Development Centre, Agriculture and Agri-Food Canada, Fredericton, Canada

**Keywords:** Chloroplast, potato, *Solanum*, plastome assembly

## Abstract

Potato (*Solanum tuberosum* L.) is the world’s fourth most important food crop and essential for global food security. The potato chloroplast genomes, the plastomes, are highly conserved and are largely studied for their maternal lineages. In this study, we assembled the complete circular plastome sequences of nine diploid potato clones, with sizes ranging between 155,296 bp and 155,564 bp. Annotation of these plastomes reveals that they each have 141 genes in a similar order. The computational chloroplast DNA typing reveals three plastid DNA types among the nine plastomes and they are grouped according to these types in the phylogeny.

The plastid DNA sequences (plastomes) of land plants are highly conserved compared to nuclear and mitochondrial DNA, and are extensively studied for their maternal phylogenetic relationships (Hosaka and Hanneman 1988). However, previous studies have shown considerable differences in plastomes and analysis of their complete sequences is important in understanding their diversity and evolutionary links (Hosaka 1986; Hosaka and Hanneman 1988; Chung et al. 2006; Achakkagari et al. 2020). In this study, a panel of nine diploid potato clones were selected for their unique characteristics such as tuber size, shape and disease resistance. Disease-free seed tubers from the following nine diploid *Solanum tuberosum* clones were obtained from the Benton Ridge Substation of Agriculture and Agri-Food Canada in Benton, New Brunswick (https://www.agr.gc.ca/eng/scientific-collaboration-and-research-in-agriculture/agriculture-and-agri-food-research-centres-and-collections/atlantic-provinces/fredericton-research-and-development-centre/?id=1180622499704) (Latitude: 45.923397 Longitude: 66.606842): *S. tuberosum* cv. 07506-01, *S. tuberosum* cv. 12120-03, *S. tuberosum* cv. DW84-1457, *S. tuberosum* cv. 12625-02, *S. tuberosum* cv. 08675-21, *S. tuberosum* cv. H412-1, *S. tuberosum* cv. W5281-2, *S. tuberosum* cv. 11379-03, and *S. tuberosum* cv. 10908-06. Potato plants were propagated in the greenhouse at the Fredericton Research and Development Centre, Fredericton NB, Canada, in six-inch clay pots with potting soil, under conditions of 16 h of light. Leaf tissue was ground and used for DNA extraction using the DNeasy Plant Mini Kit (Qiagen) according to the manufacturer’s instructions for plant tissue. Genomic DNA libraries were prepared using 10X Genomics GemCode technology (https://www.10xgenomics.com/) following the manufacturer’s instructions and whole genome *de novo* sequencing was carried out using the Novaseq 6000 DNA sequencer at the Génome Québec/Centre d’Expertise et de services Génome Québec (Montréal, Québec) at 89× coverage.

The raw reads obtained from the 10× Genomics GemCode technology were run through LongRanger Basic (https://www.10xgenomics.com/) to perform read trimming and barcode error correction. The filtered reads of each plant clone were used to assemble complete plastomes using the NOVOPlasty: *de novo* organellar genome assembler (Dierckxsens et al. 2016), which can separate plastome from nuclear reads. A seed sequence of 1000 bp was randomly selected from a *S. stenotomum* subsp. *goniocalyx* Juz. & Bukasov plastome (MT120855, CIP 702472) (Achakkagari et al. 2020). The expected genome range was set to 145–165 kbp, and other parameters set to default. The assembled sequences were annotated using GeSeq (Tillich et al. 2017) and PGA (Qu et al. 2019) with *S. stenotomum* subsp. *goniocalyx* (MT120855), *S. phureja* Juz. (MT120858, CIP 703654), *S. curtilobum* Juz. and Bukasov (MT120866, CIP 702937), and *S. tuberosum* subsp. *tuberosum* (L.) Hawkes (MT120865, CIP 705053) species as references (Achakkagari et al. 2020). The annotations were manually examined to adjust start and stop codons and to remove redundant annotations. The circular maps of these plastomes were drawn using OGDRAW v1.3.1 (Greiner et al. 2019). To understand their phylogenetic position, a maximum-likelihood phylogenetic tree was constructed using MEGA X v10.0.5 with Kimura 2-parameter substitution model (Kumar et al. 2018). The complete plastomes were used in constructing the phylogenetic tree. The chloroplast DNA types were identified by looking for specific polymorphisms that were previously identified in a panel of plastomes from a range of potato taxa (Achakkagari et al. 2020).

The assembled sizes range from 155,296 bp to 155,564 bp with the typical quadripartite structure of potato plastomes. All the nine of them have 25,593 bp of inverted repeats. The size of SSC and LSC ranges from 18,373 bp to 18,376 bp and 85,737 bp to 86,005 bp respectively. The plastomes of the W5281.2, 12625-02, 12120-03, and 11379-03 clones have the same size of 155,492 bp. Similarly, the plastomes of clones H412-1, DW84-1457, 07506-01, and 08675-21 have the same size of 155,296 bp. Overall, 141 genes are present in each plastome, with similar gene content and gene order (Figure 1). Of these, 88 are protein-coding genes, 45 are tRNAs and 8 are rRNAs. There are five basic plastid DNA types in potato that were derived through point mutations (Hosaka 1986). The DNA typing reveals three types among the nine genomes. The W5281.2, 12625-02, 12120-03, and 11379-03 genomes have the S-type, while the H412-1, DW84-1457, 07506-01, and 08675-21 genomes have the T-type plastid DNA and the 10908-06 has the W-type. The complete plastomes of these nine potato clones and the four reference plastomes used for annotation are included in the phylogeny to determine their phylogenetic relationships. It was observed that all thirteen genomes are grouped together according to their plastid DNA type.

**Figure 1.**
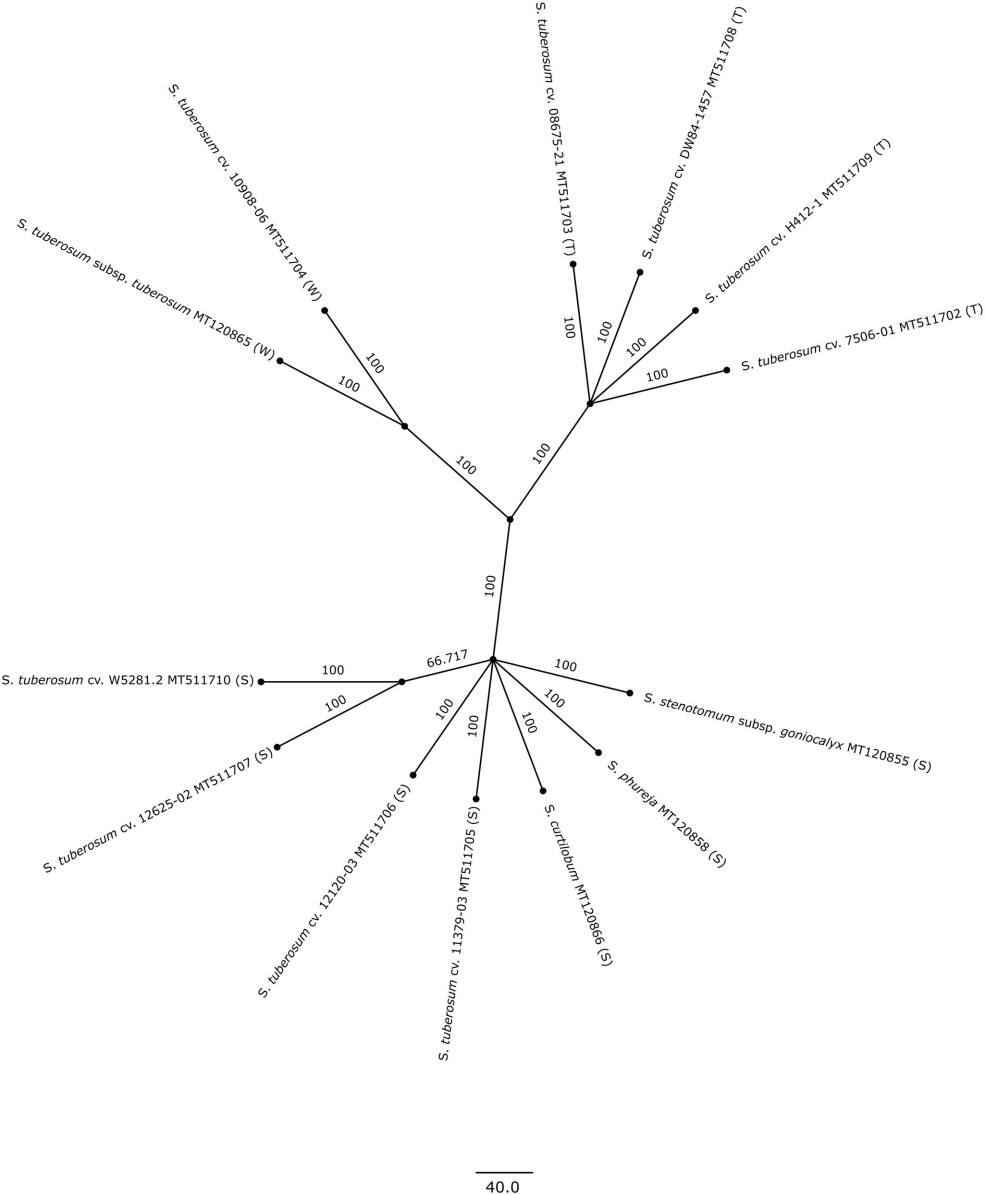
Maximum likelihood phylogenetic tree constructed using complete plastome sequences of Solanum tuberosum W5281.2 (MT511710), 12625-02 (MT511707), 12120-03 (MT511706), 11379-03 (MT511705), H412-1 (MT511709), DW84-1457 (MT511708), 07506-01 (MT511702), 08675-21 (MT511703), and 10908-06 (MT511704). The plastomes of four reference potato species, S. stenotomum subsp. goniocalyx (MT120855), S. phureja (MT120858), S. curtilobum (MT120866), and S. tuberosum subsp. tuberosum (MT120865) are also included in the phylogeny (Achakkagari et al., 2020). The plastid DNA type of each genome is given in parentheses (S, T or W).

Future studies focusing on the agronomic traits associated with the plastome types in this study can greatly facilitate breeding programs in potato. Also, determining parental origin is trivial to deal with more and more fertility problems in diploid potato breeding. The phylogeny in this study is useful in understanding the evolutionary relationships, hybridization and introgression between these species, when combined with a nuclear phylogeny.

## Data Availability

The genome sequence data that support the findings of this study are openly available in GenBank of NCBI at (https://www.ncbi.nlm.nih.gov/nuccore/) under the accession numbers MT511702-MT511710. The associated BioProject, SRA, and Bio-Sample numbers are PRJNA684565, SRR13321685-SRR13321693, and SAMN17059154-SAMN17059162, respectively.

## References

[CIT0001] Achakkagari SR, Kyriakidou M, Tai HH, Anglin NL, Ellis D, Strömvik MV. 2020. Complete plastome assemblies from a panel of 13 diverse potato taxa. PLOS One. 15(10):e0240124. 33031462 10.1371/journal.pone.0240124 PMC7544113

[CIT0002] Chung H-J, Jung JD, Park H-W, Kim J-H, Cha HW, Min SR, Jeong W-J, Liu JR. 2006. The complete chloroplast genome sequences of *Solanum tuberosum* and comparative analysis with Solanaceae species identified the presence of a 241-bp deletion in cultivated potato chloroplast DNA sequence. Plant Cell Rep. 2*5*(12):1369–1379. 16835751 10.1007/s00299-006-0196-4

[CIT0003] CIP 702472. *Solanum stenotomum* subsp. *goniocalyx.**

[CIT0004] CIP 702937. *Solanum curtilobum.*

[CIT0005] CIP 703654. *Solanum phureja.*

[CIT0006] CIP 705053. *Solanum tuberosum* subsp. *tuberosum.*

[CIT0007] Dierckxsens N, Mardulyn P, Smits G. 2016. NOVOPlasty: de novo assembly of organelle genomes from whole genome data. Nucleic Acids Res. 45(4):e18. 10.1093/nar/gkw955 PMC5389512 28204566

[CIT0008] Greiner S, Lehwark P, Bock R. 2019. OrganellarGenomeDRAW (OGDRAW) version 1.3.1: expanded toolkit for the graphical visualization of organellar genomes. Nucleic Acids Res. 47(W1):W59–W64. 30949694 10.1093/nar/gkz238 PMC6602502

[CIT0009] Hosaka K. 1986. Who is the mother of the potato? — restriction endonuclease analysis of chloroplast DNA of cultivated potatoes. Theoretical Appl Genet. 72(5):606–618. 10.1007/BF00288998 24248070

[CIT0010] Hosaka K, Hanneman RE. 1988. Origin of chloroplast DNA diversity in the Andean potatoes. Theoretical Appl Genet. 76(3):333–340. 10.1007/BF00265332 24232196

[CIT0011] Kumar S, Stecher G, Li M, Knyaz C, Tamura K. 2018. MEGA X: molecular evolutionary genetics analysis across computing platforms. Mol Biol Evol. 35(6):547–549. 10.1093/molbev/msy096 PMC5967553 29722887

[CIT0012] Qu X-J, Moore MJ, Li D-Z, Yi T-S. 2019. PGA: a software package for rapid, accurate, and flexible batch annotation of plastomes. Plant Methods. 15(1):50. 31139240 10.1186/s13007-019-0435-7 PMC6528300

[CIT0013] Tillich M, Lehwark P, Pellizzer T, Ulbricht-Jones ES, Fischer A, Bock R, Greiner S. 2017. GeSeq – versatile and accurate annotation of organelle genomes. Nucleic Acids Res. 45(W1):W6–W11. 28486635 10.1093/nar/gkx391 PMC5570176

